# DDX21 Interacts with WDR5 to Promote Colorectal Cancer Cell Proliferation by Activating CDK1 Expression

**DOI:** 10.7150/jca.69216

**Published:** 2022-02-28

**Authors:** Peifen Lu, Zenong Yu, Kangning Wang, Yongping Zhai, Bing Chen, Ming Liu, Peipei Xu, Feng Li, Quan Zhao

**Affiliations:** 1 The State Key Laboratory of Pharmaceutical Biotechnology, Department of Hematology, the Affiliated Drum Tower Hospital of Nanjing University Medical School, China-Australia Institute of Translational Medicine, School of Life Sciences, Nanjing University, Nanjing, China.; 2 Department of Hematology, Jinling Hospital, School of Medicine, Nanjing University, Nanjing, China.

**Keywords:** DDX21, WDR5, CDK1, H3K4me3, Colorectal Cancer

## Abstract

DEAD-box RNA helicase 21 (DDX21), is a nucleolar protein harboring ATP-dependent double-stranded RNA unwinding activities, essential in rRNA processing and ribosome biogenesis. However, its role in colorectal cancer (CRC) progression remains unclear. In this study, we show that knockdown of DDX21 significantly inhibited CRC cell proliferation and blocked cell cycle at the G2/M phase. Gene profile analysis and ChIP assays revealed that DDX21 activated CDK1 gene expression through binding to the gene promoter. In addition, we found that DDX21 directly recruited WDR5 to enhance trimethylation of histone H3 on Lys 4 (H3K4me3) on the CDK1 promoter. Importantly, elevated expression of DDX21 in CRC patients was positively correlated with expression of CDK1, and these CRC patients had shorter overall survival. These findings reveal a critical novel role of DDX21 in transcriptional and epigenetic control of CRC cell proliferation. Taken together, this study uncovers that DDX21 interacted with WDR5 to promote colorectal cancer cell proliferation by activating CDK1 expression, suggesting that targeting DDX21 may be an alternative new strategy for CRC treatment.

## Introduction

Colorectal cancer (CRC) is the third most common cancer and the second leading cause of cancer-associated death worldwide 1. Although increasing levels of new treatments have doubled overall survival for the advanced disease to three years, survival is hardly improved for those high-risk CRC patients 2. Moreover, the detailed mechanisms of CRC development are still not completely understood, which makes it more difficult to decipher the development and progression of CRC and to choose the appropriate treatment for patients. Therefore, to understand the pathogenesis of CRC would not only help explore new molecular biomarkers and candidate therapeutic targets, but also help develop precision treatment methods for CRC patients.

DEAD-box RNA helicase 21 (DDX21), a member of the DExD/H box family, has ATP-dependent double-stranded RNA unwinding and ATP-independent G-quadruplex remodeling activities in homodimeric form 3. DDX21 is a multitasking enzyme, plays vital roles in ribosomal RNA biogenesis 4, transcription, RNA metabolism 3-8 and the immune response during virus infection 9-12, especially in promoting tumorigenesis 13-17. Thus, DDX21 has been found to interact with a variety of protein partners, including XRN2 (a snoRNA-processing exonuclease), Ago (small RNAs guide Argonaute, the main RISC component), JMJD3 (a Lysine Demethylase), WDR46 (a nuclear scaffold protein) 18-21. DDX21 is highly expressed in breast cancer 22, colorectal cancer 14, 23, gastric cancer 17, neuroblastoma 13 and melanoma 15. In gastric cancer, upregulated DDX21 increased cell proliferation *in vitro* and tumor progression in mice via the Cyclin D1 and CDK2 pathways 17. In contrast, DDX21 was found to act on Snail gene promoter and epigenetically repressed its transcription resulting in suppression of epithelial-mesenchymal transition and invasion of breast cancer cells 20. However, there are few reports about the role of DDX21 in colorectal cancer, although DDX21 has been identified as a candidate biomarker with high confidence 23. Therefore, the complex roles of this multifunctional protein DDX21 await to be explored in colorectal cancer.

ChIP-seq analysis found that DDX21 tends to bind to trimethylation of lysine 4 of histone 3 (H3K4me3)-enriched promoters, a histone hallmark for transcription initiation 4. However, the underlying mechanism is unknown. H3K4me3 mark is deposited by the SET domain containing 1 (SET1)/Mixed Lineage Leukemia (MLL) histone methyltransferase complexes, and one of the core components of this complex is WD repeat domain 5 (WDR5) 24, 25. WDR5 is a highly conserved WD40 repeat-containing protein, and is essential for the proper regulation of multiple cellular processes 26. WDR5 can form protein complexes with transcription factors to regulate gene transcription. Recent studies have revealed that WDR5 plays key roles in the tumorigenesis and progression of a variety of cancers, such as breast cancer 27, neuroblastoma 28, pancreatic cancer 29, prostate cancer 30, gastric cancer 31 and non-small cell lung cancer 32, 33. However, similarly, its roles in colon cancer are less understood.

In this study, we show that DDX21 interacted directly with WDR5 to promote CRC cell proliferation. DDX21 activated CDK1 gene expression concurrent with deposition of histone mark H3K4me3 on CDK1 promoter. These results provide cellular evidence that DDX21 could be a potential novel therapeutic target for CRC.

## Material and methods

### Cell culture

HEK293T, HCT8 and SW620 cells were purchased from the National Collection of Authenticated Cell Cultures (Shanghai, China). HCT8 and SW620 cells were cultured in RPMI-1640 medium, while HEK293T cells were cultured in Dulbecco's modified Eagle's medium (DMEM) medium, supplemented with 10% FBS (Excell, Shanghai) and 1% penicillin-streptomycin (Beyotime Biotechnology, Beijing). All cell lines were cultured in a humidified atmosphere of 5% CO_2_ and 95% air at 37 °C and were routinely tested to exclude mycoplasma contamination, authenticated using short tandem repeat (STR) profiling.

### Plasmid construction, RNAi treatment and lentiviral transduction

pLVX-Flag-DDX21-puro was constructed by cloning the coding sequence of DDX21 from cDNA of HEK293T into the XhoI and BamHI sites of pLVX-IRES-puro (MiaoLing Plasmid Sharing Platform, Hubei) and Flag tag was added into its N-terminal by PCR. pGEX-6p-1-DDX21 was subcloned from pLVX-Flag-DDX21-puro into the BamHI and XhoI sites. pGEX-WDR5 was subcloned from MSCV-3HA-WDR5-IRES-GFP 34 into the EcoRI and XhoI sites of pGEX-6p-1. pLKO.1-TRC was purchased from Addgene. shRNA targeting sequence or scramble sequence were cloned into pLKO.1-TRC vector with AgeI and EcoRI. The DDX21-targeting sequences were: shDDX21-1: 5'-CCTGAGGTTGATTTGGTTATA-3'; shDDX21-2: 5'-CCCATATCTGAAGAAACTATT-3'; Scrambled sequences: 5'-CCTAAGGTTAAGTCGCCCTCG-3'. All constructs were confirmed by DNA sequencing.

siRNA was synthesized by Nanjing KeyGen Biotech (Nanjing, China). The WDR5-targeting sequences were: siWDR5-1: GTGGAAGAGTGACTGCTAA, siWDR5-2: GAATGAGAAATACTGCATA. siRNA transfection was performed according to the manufacturer's instructions. Lentiviral infections were performed by transfecting HEK293T cells with lentiviral constructs and packaging plasmids (psPAX2 and pMD2.G) by DNAfectin^TM^ Plus Transfection Reagent (Applied Biological Materials, Canada). Viral supernatants were collected 48 hours after transfection. Cells were infected with lentivirus and selected for 5 days in 4 μg/ml puromycin.

### RNA extraction and quantitative reverse transcriptase-PCR (qRT-PCR)

Total RNA was extracted using TRIzol reagent (Invitrogen) and was reverse transcribed into cDNA using HiScript Q RT SuperMix kit (Vazyme Biotech, Nanjing) according to the manufacturer's instructions. qRT-PCR analysis was performed using SYBR green qPCR master mix (Vazyme Biotech, Nanjing) with the Applied Biosystems 7500 System (Applied Biosystems, USA). GAPDH was used as the normalized control. Fold expression change was analyzed using ΔΔCT methods. The primer sequences for qRT-PCR are listed in Table S1.

### RNA-Seq and data analysis

RNA-Seq was performed and analyzed by Shanghai Jiayin Biotechnology Co., Ltd. RNA-seq transcriptome library was prepared with random hexamer primers (Illumina) according to Illumina's library construction protocol. Sequencing was performed on the Illumina Novaseq6000 (2 × 150 cycles per base). After Quality Control by FastQC, adapter sequences and poor-quality reads were removed using Cutadapt. All quality-filtered reads were mapped to the human genome through STAR, and subjected to a series of bioinformatics analyses and plotting, including Gene Expression Analysis, Principal Component Analysis, Different Gene Analysis, Heatmap Plot, Gene Ontology Analysis, Pathway Analysis and Gene Set Enrichment Analysis. The datasets of RNA-seq have been submitted to the GEO databases under accession number GSE184726.

### Western blot, Coimmunoprecipitation assay (Co-IP) and Chromatin immunoprecipitation (ChIP) assay

Cells were lysed in RIPA lysis buffer (Beyotime, Beijing) according to the manufacturer's instructions. Protein extracts were separated by SDS-PAGE and electrophoretically transferred to PVDF membranes (Roche, Switzerland). Membranes were blocked in 5% BSA/PBST at room temperature for one hour and incubated with primary antibodies overnight. Immunoblots were detected with the Tanon^TM^ High-sig ECL Western blotting substrate (Tanon, Shanghai). The information of the primary antibodies is listed in Table S2. For the coimmunoprecipitation assay, cell lysates were prepared using RIPA lysis buffer (Beyotime, Beijing). Whole cellular extracts were immunoprecipitated with ANTI-FLAG M2 Affinity Gel (Sigma-Aldrich, USA) (for exogenous binding) or anti-DDX21 antibody (for endogenous binding), or isotype IgG (as a control) (Cell Signaling, USA) with Protein A resin (GeneScript, Nanjing) overnight at 4 °C. Chromatin immunoprecipitation was performed as described previously^35^. ChIP-primer sequences are listed in the Table S3.

### Cell cycle analysis, cell proliferation and colony formation assay

Samples for cells cycle analysis were prepared using Cell Cycle Detection Kit (KeyGEN BioTECH, Nanjing) according to the manufacturer's instructions. Cells were subsequently analyzed by Attune NxT Flow Cytometer (Thermo Fisher, American). Cell populations were calculated using the FlowJo V10 software.

For cell proliferation assay, cells were seeded onto 96-well plates (2000 cells per well) and measured by CCK-8 cell counting kit (Vazyme Biotech, Nanjing) according to the manufacturer's instructions.

For colony formation assay, cells were seeded in 6-well plates (1000 cells per well) and cultured for 10 days, and then fixed with methanol and stained with 0.1% crystal violet.

### Protein expression, purification and GST pull-down assay

All recombinant proteins were produced in *Escherichia coli* BL21 (DE3). After bacteria were grown to an OD of 0.6, protein expression was induced with 0.5 mM IPTG at 37°C for 4 h. Bacterial pellet was resuspended in lysis buffer (25mM Tris-HCl, 125mM NaCl, 1% TritonX-100, 0.2 mM PMSF, pH8.0), sonicated and centrifuged at 10000g at 4°C for 30 min. The soluble recombinant protein was enriched by GST resin (GeneScript, Nanjing). GST Resin were washed by NETN (300mM/500mM NaCl, 1mM EDTA·2Na, 50mM Tris-HCl, 0.5% NP-40, pH 8.0) and eluted by GSH elution buffer (20mM glutathione, 50mM Tris-HCl, 100mM NaCl, pH 7.5). GST-tag cleavage was performed using PreScission Protease (GeneScript, Nanjing) according to the manufacturer's instructions. Protein purity and quantity were assessed by SDS-PAGE following Coomassie staining. For GST pull down assay, the equal amount of GST or GST-fusion protein and tested protein were mixed and incubated for 2~4 hours at 4 °C for binding reactions before interacting proteins were subsequently harvested by GST resin (GeneScript, Nanjing) washed by NETN 500 (500mM NaCl, 1mM EDTA·2Na, 50mM Tris-HCl, 0.5% NP-40, pH 8.0) three to six times and then subjected to Western blotting.

### Statistical analysis

Statistical analyses were determined by Student's *t*-test and performed by GraphPad Prism software V 6.02. All data were shown as the mean ± SD of more than three independent experiments. *P* < 0.05 was considered to indicate a statistically significant difference.

## Results

### Knockdown of DDX21 reduces CRC cell proliferation and arrests cell cycle

**Figure 1:**
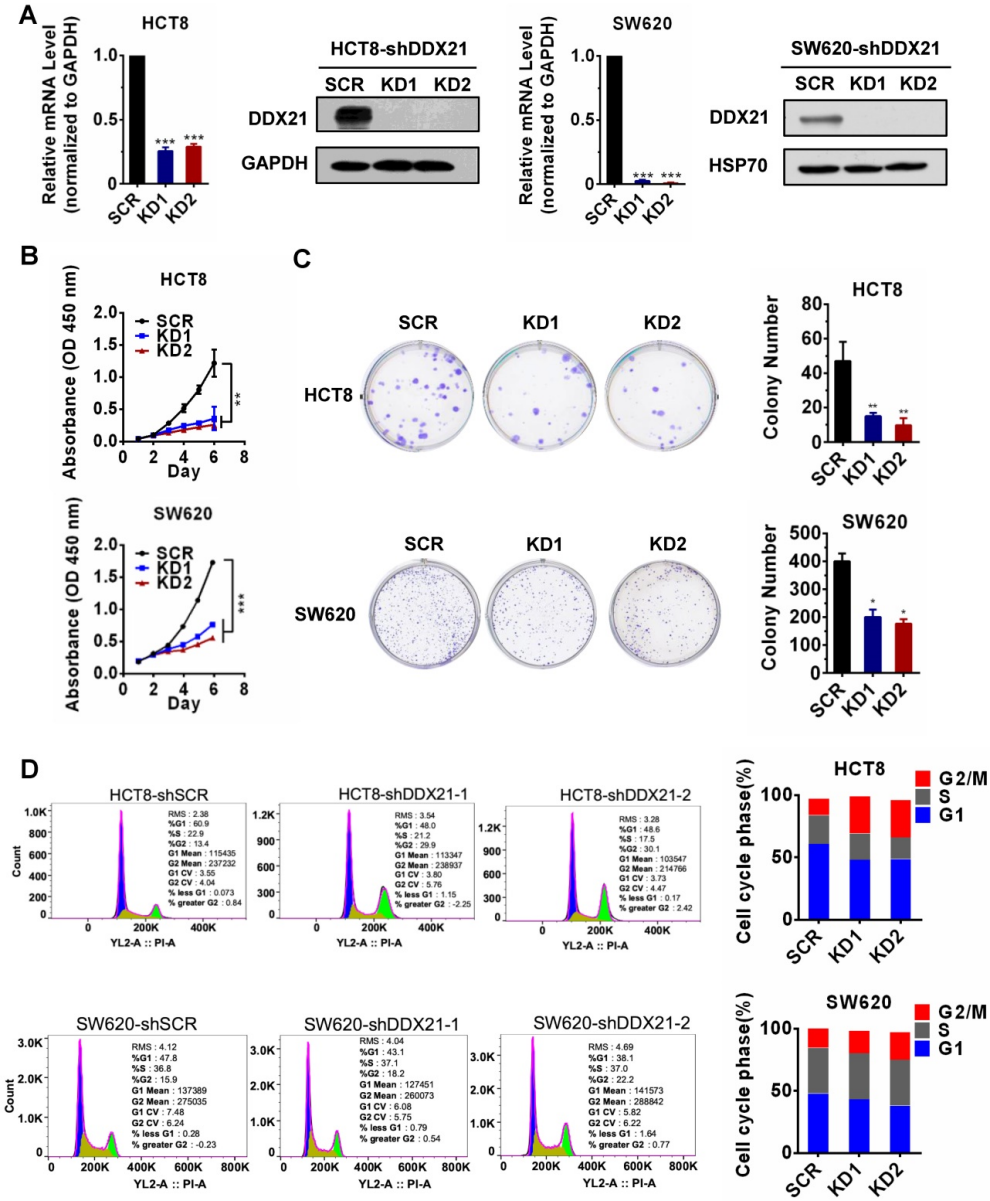
Knockdown of DDX21 reduces CRC cell proliferation and arrests cell cycle. (A) DDX21 was efficiently knocked down in HCT8 and SW620 cells by RNA interference. (B) (C) DDX21 silencing inhibits cell growth and colony formation ability. (D) DDX21 knocking down induces the G2/M arrest in CRC cells. SCR, scrambled control. KD1, shDDX21-1. KD2, shDDX21-2. All experiments were performed in triplicate. * *P* < 0.05; **P < 0.01; ***P < 0.001.

Data from The Cancer Genome Atlas (TCGA) database and other studies showed that DDX21 was aberrantly upregulated in CRC patients and that those patients had shorter overall and disease-free survival (Figure S1A-D) 14. However, the role of DDX21 during the progression of CRC remains unclear. In order to evaluate the effect of DDX21 on CRC cell growth, we knocked down DDX21 expression in two CRC cell lines, HCT8 cells and SW620 cells independently. As expected, qRT-PCR and Western blot analyses showed that the mRNA and protein levels of DDX21 were markedly decreased in both knockdown HCT8 cells and SW620 cells compared to SCR cells (Figure 1A). We observed a significant slower cell growth in DDX21 knockdown cells compared to SCR cells from either cell proliferation CCK-8 assay or colony cell formation assay (Figure 1B, C). In contrast, we found no difference in numbers of apoptotic cells between DDX21 knockdown and SCR cells (Figure S2). In addition, we performed flow cytometry analysis to investigate the influence of DDX21 knockdown on cell cycle. We found that there were significant more DDX21 knockdown cells arrested at G2/M phase compared to SCR cells, suggesting that DDX21 may associate with cell cycle regulation (Figure 1D). These results indicate that DDX21 promoted CRC cell proliferation and may be involved in cell cycle regulation.

### DDX21 regulates the transcription of cell cycle-related genes in CRC cells

**Figure 2:**
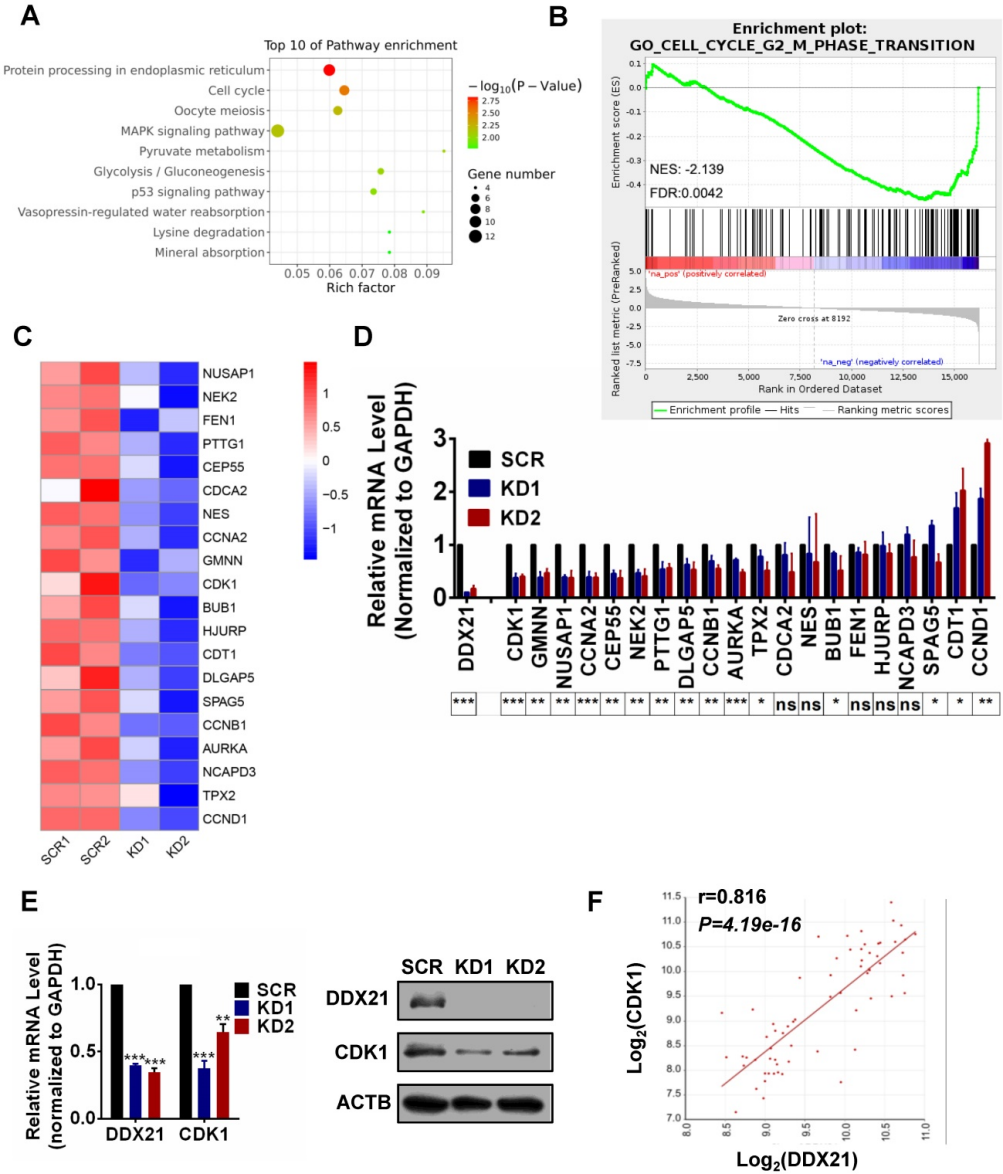
Identification of DDX21 downstream target genes in CRC cells. (A) Pathway analysis shows top 10 enrichment for all genes with altered expressions. (B) Genes altered expressions are correlated with the cell cycle transition. (C) Heatmap of top 20 downregulated genes related cell cycle. (D) The mRNA levels of select genes quantitated by qRT-PCR in DDX21 knocking down HCT8 cells. *P* values are indicated on the bottom of the bar graph. (E) qPCR and western blot show that DDX21 knocking down represses the expression of CDK1. (F) Correlations between DDX21 and CDK1 in CRC patients (GSE8671). * *P* < 0.05; **P < 0.01; ***P < 0.001; ns, not signification.

To explore how DDX21 functions in cell cycle in CRC cells, we performed RNA-Seq analysis in DDX21 knockdown and SCR HCT8 cells. A total of 307 genes differentially expressed genes between DDX21 KD and SCR cells were identified (*P*<0.05, |Log_2_Fold Change|>0) (Figure S3A). Pathway and GO analysis showed that DDX21 knockdown related genes were preferentially involved in protein processing, mitotic nuclear division, and cell cycle (Figure 2A, Figure S3B). To identify molecular pathways potentially associated with DDX21, we performed gene set enrichment analysis (GSEA). GSEA results revealed a significant enrichment of signature genes particularly associated with cell cycle G2/M phase transition, consistent with the effect of DDX21 knockdown on CRC cells (Figure 2B). To gain further insights into DDX21 knockdown-related pathway in cell cycle blocking, we focused on the top 20 downregulated genes from RNAseq analysis highly associated with cell cycle pathways (Figure 2C). Among these 20 genes, qRT-PCR analysis confirmed that CDK1 expression was the most markedly decreased at the mRNA level in DDX21 knockdown cells compared to SCR cells (Figure 2D). Consistently, protein levels of CDK1 were also significantly decreased in DDX21 knockdown cells compared to SCR cells (Figure 2E). More importantly, the expression levels of CDK1 correlated well with the expression levels of DDX21 in CRC samples (Figure 2F). Taken together, these results suggest that DDX21 could transcriptionally regulate CDK1 expression, likely for maintenance of cell cycle in CRC.

### DDX21-knockdown decreases CDK1 expression to inhibit cell proliferation

**Figure 3:**
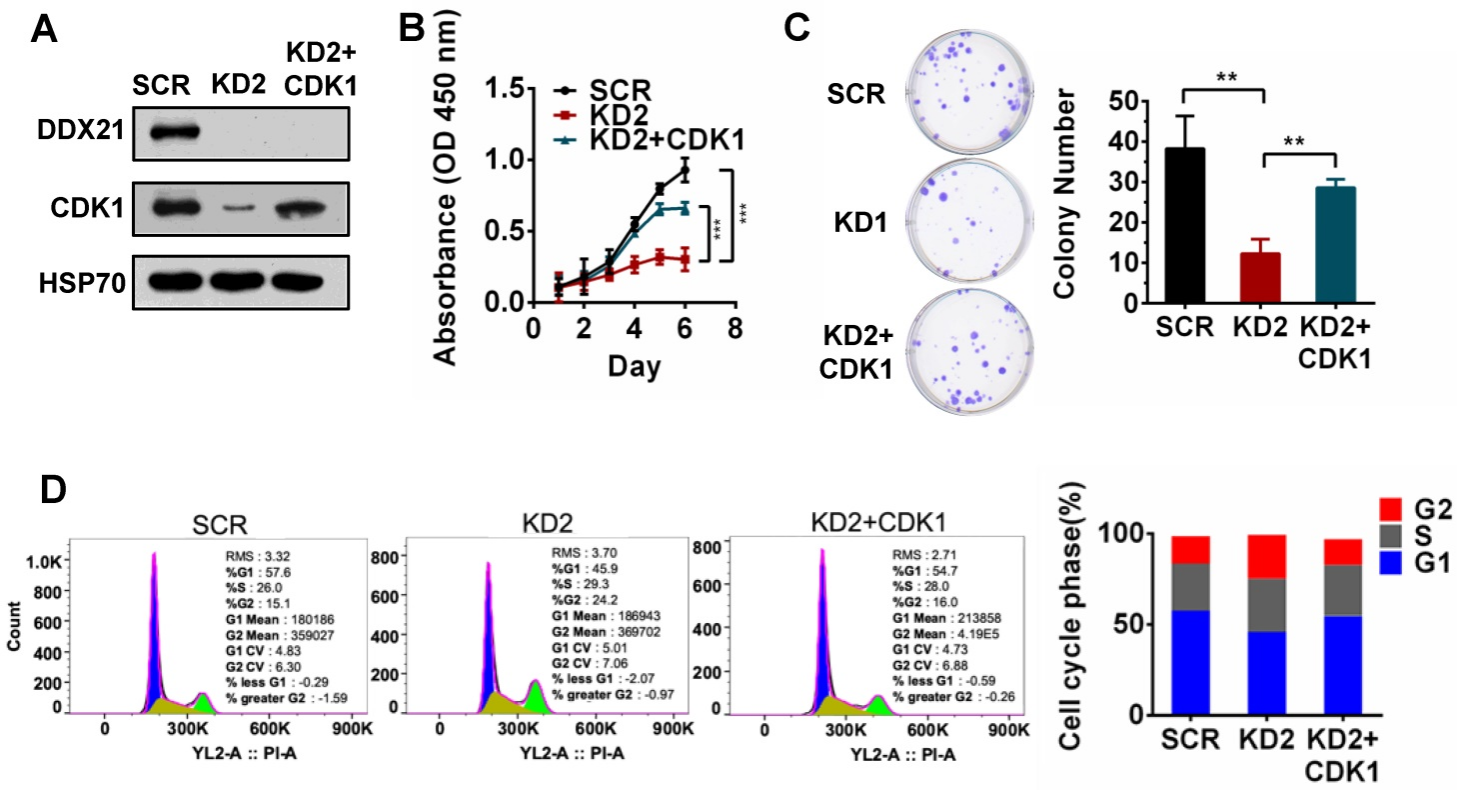
DDX21-knockdown decreases CDK1 expression to inhibit cell proliferation. (A) Western blot shows the protein level of CDK1 is rescued by exogenous CDK1 expression. (B-D) Rescuing CDK1 partially reverses cell proliferation by CCK8, colony formation and the G2 / M phase cell numbers. All experiments were performed in triplicate. * *P* < 0.05; **P < 0.01; ***P < 0.001.

To validate whether CDK1 is a downstream gene regulated by DDX21 during cell proliferation, we tested the effect of CDK1 overexpression in DDX21-knockdown CRC cells. We first confirmed the overexpression of CDK1 in DDX21 knockdown CRC cells by western blot analysis (Figure 3A). Later on, we found that overexpression of CDK1 could reverse the effect of DDX21-knockdown on cell proliferation, colony formation and cell cycle arrest at the G2/M stage (Figure 3B-D). These results indicated that CDK1 is one of the key downstream genes regulated by DDX21 during CRC cell proliferation.

### DDX21 regulates CDK1 gene expression by directly binding to its promoter

**Figure 4:**
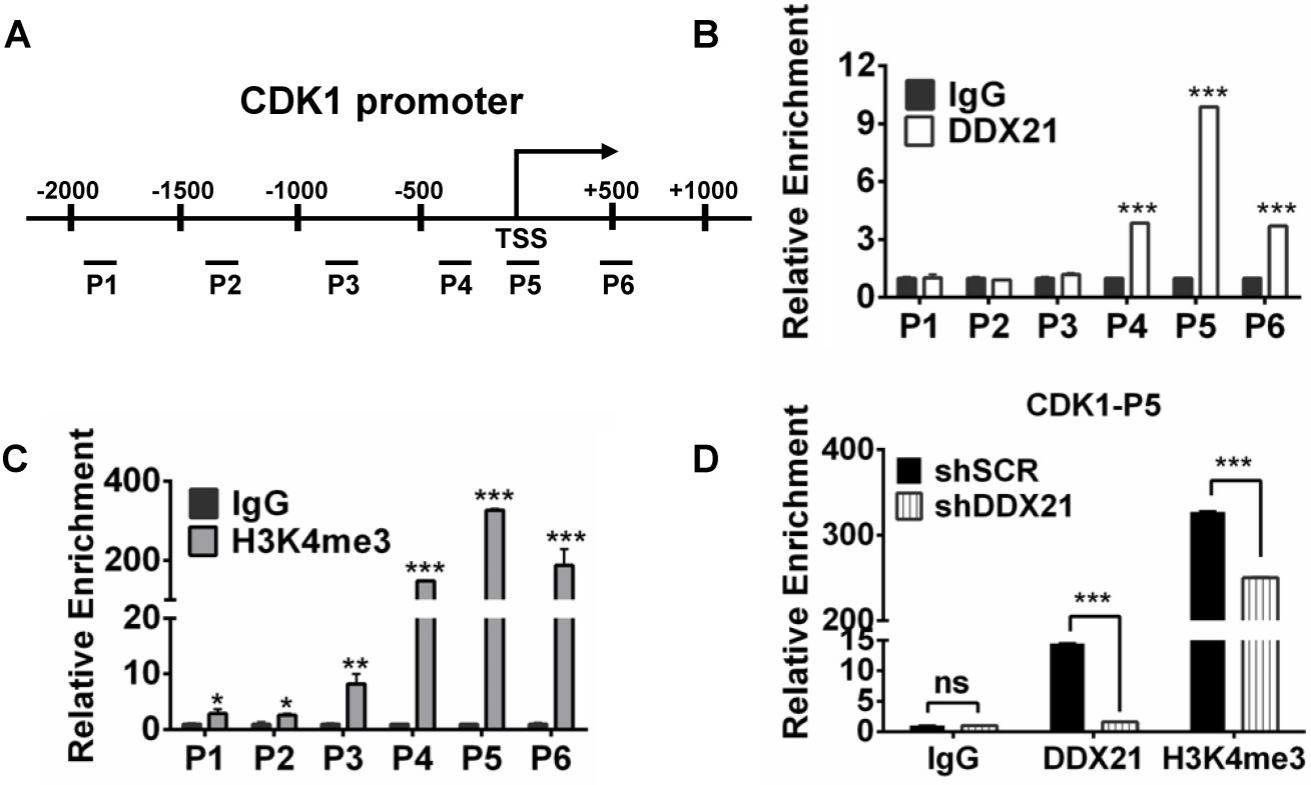
DDX21 regulates CDK1 gene expression by directly binding to its promoter. (A) Schematic representation of ChIP assay of HCT8 cells using walking primers (positions P1-P6 relative to TSS) at the CDK1 promoter. P1: -1996 to -1842, P2: -1411 to -1299, P3: -946 to -787, P4: -407 to -254, P5: -94 to +103, P6: +446 to +594. TSS, transcription start site. (B, C) ChIP-qPCR analysis of DDX21 and H3K4me3 of HCT8 of the CDK1 promoter. (D) ChIP-qPCR analysis of H3K4me3 levels at the CDK1 promoter after knocking down DDX21. Enrichment fold were calculated relative to IgG. All experiments were performed in triplicate. * *P* < 0.05; **P < 0.01; ***P < 0.001.

To explore how DDX21 regulates the expression of CDK1, we designed six pairs of walking primers on the CDK1 gene promoter and performed ChIP experiments to test whether DDX21 binds on the CDK1 gene promoter. We found that DDX21 could be significantly enriched at the CDK1 promoter P5 region compared with IgG controls (Figure 4A, B). Previous ChIP-seq analysis revealed that DDX21-bound promoters had high enrichment of gene activation mark, H3K4me3 4. Given that DDX21 positively regulates CDK1 gene expression, we reasoned whether the histone H3K4me3 mark was involved in this process. Therefore, we performed ChIP analysis with H3K4me3 antibody, and found that H3K4me3 was indeed the most enriched in the P5 region on the CDK1 promoter, which coincided with the binding region of DDX21 (Figure 4C). To confirm this finding, we knocked down DDX21 in HCT8 cells, and performed the same ChIP analysis again. We showed that the levels of DDX21, as well as H3K4me3 enrichment, were both markedly decreased in DDX21 knockdown cells compared to SCR cells (Figure 4D). These results show that DDX21 regulated CDK1 gene expression by directly binding to its promoter.

### DDX21 interacts with WDR5 to regulate CDK1 expression

**Figure 5:**
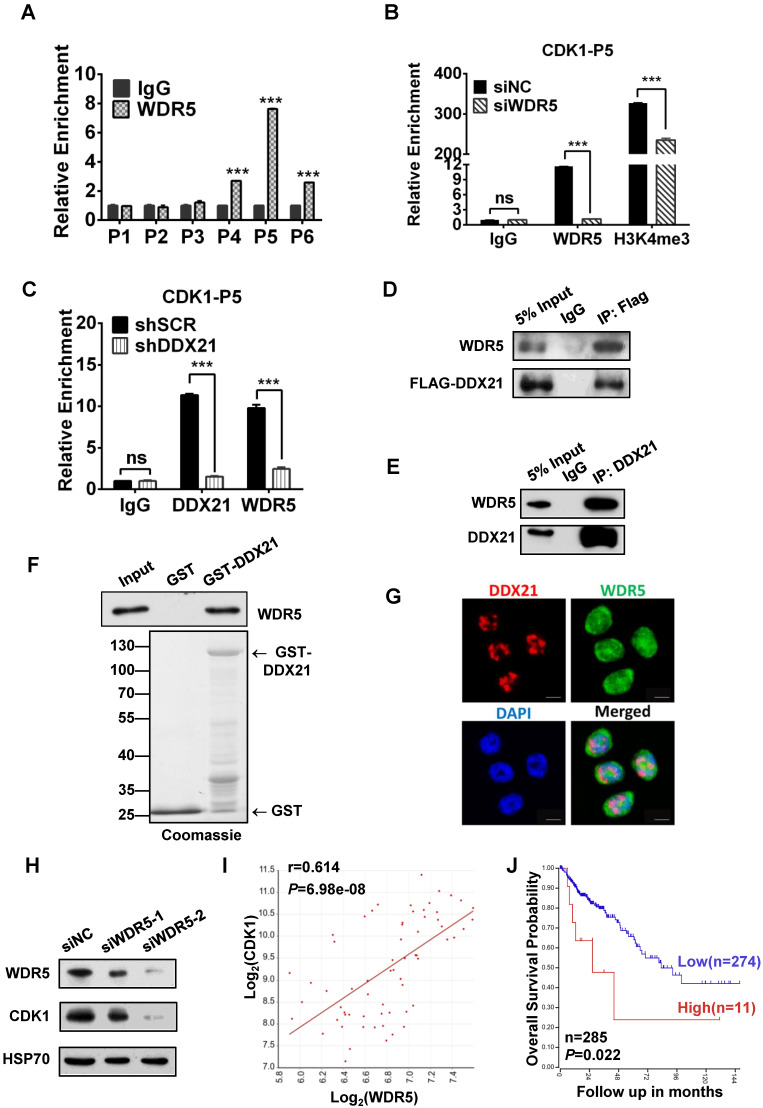
DDX21 interacts with WDR5 to regulate CDK1 expression. (A) ChIP-qPCR shows that WDR5 binds to the CDK1 promoter. (B) The deposition of H3K4me3 at the CDK1 promoter is decreased by depleting WDR5. (C) ChIP-qPCR analysis of DDX21, and WDR5 levels at CDK1 promoter after depleting DDX21. (D) Western blot showing exogenous expression of Flag-tagged DDX21 and immunoprecipitated WDR5 in HCT8 lysates. (E, F) DDX21 and WDR5 interaction is confirmed by endogenous co-IP and *in vitro* GST-pulldown assays. (G) The subcellular location of DDX21 and WDR5 proteins was documented in HCT8 cells by immunofluorescence microscopy. Scale bar: 10 μm. (H) Western blot shows that the expression of CDK1 is downregulated by WDR5 knocking down. (I) Correlations between expression of WDR5 and CDK1 in CRC patients (GSE8671). (J) Increased WDR5 mRNA levels were correlated with poor overall survival in colon cancer patients from TCGA Tumor Colon Adenocarcinoma patient cohort. P1-P6, CDK1 promoter walking primers described in Figure 4A. All experiments were performed in triplicate. * *P* < 0.05; **P < 0.01; ***P < 0.001.

Since WDR5 is a core subunit of the MLL or SET1 methyltransferase complex which tri-methylated histone H3K4, and is required for complex assembly and methyltransferase activity, we tested whether WDR5 is involved in the process of modulation of the H3K4me3 levels on the CDK1 promoter. Thus, we performed ChIP analysis with WDR5 antibody, and found that WDR5 was also most enriched in the P5 regions on the CDK1 promoter, which overlaps with the binding region of DDX21 (Figure 5A). In addition, we showed that the levels of H3K4me3 enrichment were significantly decreased in WDR5 knockdown cells compared to SCR cells, indicating a positive influence of WDR5 on H3K4me3 on the CDK1 promoter (Figure 5B). Furthermore, we observed that knocking down of DDX21 resulted in a marked reduction of WDR5 on CDK1 promoter (Figure 5C), which suggested that DDX21 may regulate the CDK1 expression through WDR5 to control the modification of H3K4me3 on its promoter.

Based on these results, next, we wanted to check whether WDR5 physically interacts with DDX21 to regulate CDK1 gene expression. Coimmunoprecipitation experiments of endogenous or exogenous proteins from the lysates of HCT8 cells showed that DDX21 could be immunoprecipitated with WDR5 in HCT8 cells (Figure 5D, E). Furthermore, GST pull-down assays showed that DDX21 could directly interact with WDR5 *in vitro* (Figure 5F). In addition, we found that DDX21 was co-localized with WDR5 dominantly in the nucleus of HCT8 cells (Figure 5G). To confirm the role of WDR5 in regulating CDK1 expression, we knocked down WDR5 expression in HCT8 cells, and found the expression of CDK1 was significantly reduced compared to SCR cells (Figure 5H). Consistently, transcriptome analysis results showed that the expression levels of CDK1 correlated well with those of WDR5 in colorectal adenomas (GSE8671) (Figure 5I). More importantly, data from the TCGA database showed that WDR5 is aberrantly upregulated in CRC patients and that those patients had shorter overall survival (Figure 5J, Figure S4). Together, these results indicate that DDX21 interacts with WDR5 to regulate CDK1 expression in CRC cells.

## Discussion

Although DDX21 has been considered as a potent prognosis marker for CRC 14, 23, the role of DDX21 in CRC development is unclear. In this study, we found that DDX21 promoted the proliferation of CRC cells through activating the expression of CDK1 to accelerate the cell cycle. We showed that DDX21 interacted directly with WDR5 to deposit H3K4me3 mark on the CDK1 promoter, thus enhancing CDK1 expression at the transcription level. Our results demonstrate a novel role for DDX21 in controlling CRC proliferation and suggest a potential novel therapeutic target in CRC.

DDX21 has been demonstrated to act as an oncoprotein and is highly expressed in multiple cancers 13-17. For instance, DDX21 was reported to induce CEP55 expression, MYCN-amplified neuroblastoma cell proliferation, and tumorigenesis 13. DDX21 was also found to promote gastric cancer cell growth by up-regulating levels of Cyclin D1 and CDK2 17. However, the role of DDX21 in CRC remained to be elucidated. Herein, we showed that DDX21 was highly expressed in different stages of CRC and correlated with poor prognosis, which is consistent with the results obtained by Tanaka A et al. 14. We found that DDX21 depletion inhibited CRC cell proliferation and disrupted the G2/M checkpoint, which agrees with the observation in CRC cell lines SW480 and SW620 36. Notably, we found that DDX21 knockdown significantly decreased the expression of CDK1 to halt the cell cycle which was further supported by our rescue experiments. Our results provided a novel insight into how DDX21 may function in CRC cell proliferation and development.

Epigenetic regulators play important roles and contribute to the pathological processes of many cancers. It has been reported that DDX21 interacted with H3K27me3 demethylase JMJD3/KDM6B, and was recruited to the transcription start site to regulate the expression of ENPP2 19, suggesting that DDX21 may interact with epigenetic regulators to form a complex to regulate specific subsets of genes. In this study, we found that DDX21 directly interacted with WDR5, the core component of the MLL/SET1 complex, which triggers trimethylation of H3K4. WDR5 was highly expressed in many cancers, and overexpression of WDR5 is associated with advanced tumor stage and poor patient survival rate. WDR5 was found to promote cell proliferation through inducing H4K3me3 on the Cyclin D promoter to activate its expression in gastric cancer 37. In lung cancer and breast carcinoma cells, WDR5 could recruit protein arginine methyltransferase 5 (PRMT5) complexes to target gene promoters, enhancing the deposition of H3K4me3 to promote gene transcription and cancer cell invasion 38. These results support a relationship between WDR5 and tumorigenicity. In addition, WDR5 was shown to have a multitude of interactions participating in diverse complexes such as transcription factor MYC, MLL Complex, NSL (non-specific lethal) Complex, or NuRD Complex 39. In this study, we found a new WDR5 interactor, DDX21, an RNA helicase, which further supports the complexity of the WDR5 functions. Furthermore, our results are consistent with the ChIP-seq data which showed high DDX21-bound promoters harboring a high enrichment of H3K4me3 4, and reveal the new link between DDX21, WDR5 and histone mark H3K4me3. Considering WDR5 is a promising target for pharmacological inhibition in cancer 40, WDR5 inhibitor may also be feasible for CRC patients.

In conclusion, our data illustrated that DDX21 promoted CRC cell proliferation via recruiting WDR5 to transcriptionally activate the expression of CDK1. Based on these findings, we propose that silencing DDX21 expression could represent a novel strategy for CRC therapy.

## Supplementary Material

Supplementary figures and tables.

## Abbreviations

DDX21: DEAD-box RNA helicase 21
CRC: colorectal cancer
H3K4me3: trimethylation of histone H3 on Lys 4
WDR5: WD repeat domain 5
Co-IP: Coimmunoprecipitation assay
ChIP: Chromatin immunoprecipitation
TCGA: The Cancer Genome Atlas
