# Supplementary material for: DDX21 Interacts with WDR5 to Promote Colorectal Cancer Cell Proliferation by Activating CDK1 Expression
Source: J Cancer. 2022 Feb 28;13(5):1530–9. doi: 10.7150/jca.69216 (PMC8965128; doi:10.7150/jca.69216)
Supplement: Supplementary file 1 — Supplementary figures and tables. [file jcav13p1530s1.pdf]

Supplemental materials

DDX21 Interacts with WDR5 to Promote Colorectal Cancer Cell Proliferation by Activating CDK1 Expression

Peifen Lu[1], Zenong Yu[1], Kangning Wang[1], Yongping Zhai[2], Bing Chen[1], Ming Liu[1], Peipei Xu[1,*], Feng Li[2,*], Quan Zhao[1,*]

[1]The State Key Laboratory of Pharmaceutical Biotechnology, Department of Hematology, the Affiliated Drum Tower Hospital of Nanjing University Medical School, China-Australia Institute of Translational Medicine, School of Life Sciences, Nanjing University, Nanjing, China; [2]Department of Hematology, Jinling Hospital, School of Medicine, Nanjing University, Nanjing, China.

[*]Corresponding Authors

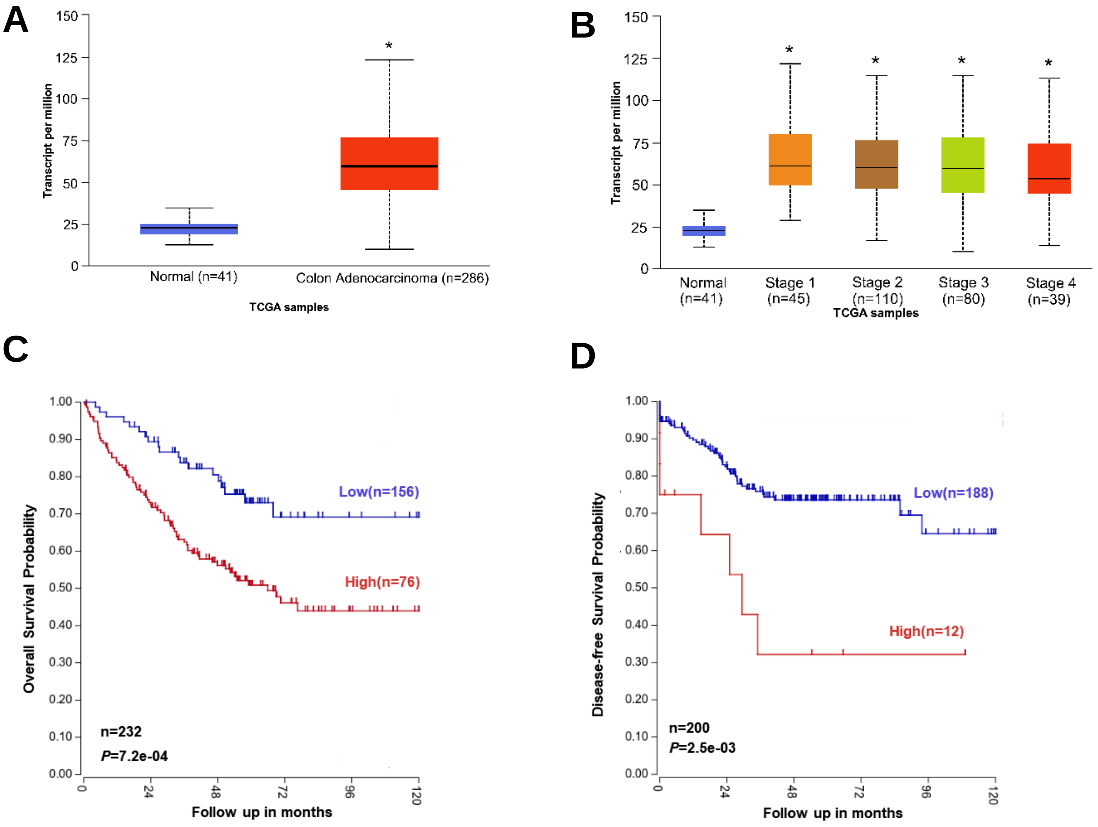

Figure. S1 DDX21 is overexpressed in CRC and correlated with poor prognosis. A, DDX21 is highly expressed in colon adenocarcinoma compared with normal tissues. B, DDX21 is overexpressed from stage 1 to stage 4 of colon adenocarcinoma. Analysis was performed by UALCAN. C, D, Overall survival and progression-free survival curves indicates that CRC patients with high DDX21 expression have obviously lower overall survival rates than those with low DDX21 expression (GSE17538). *$P < 0.05$.

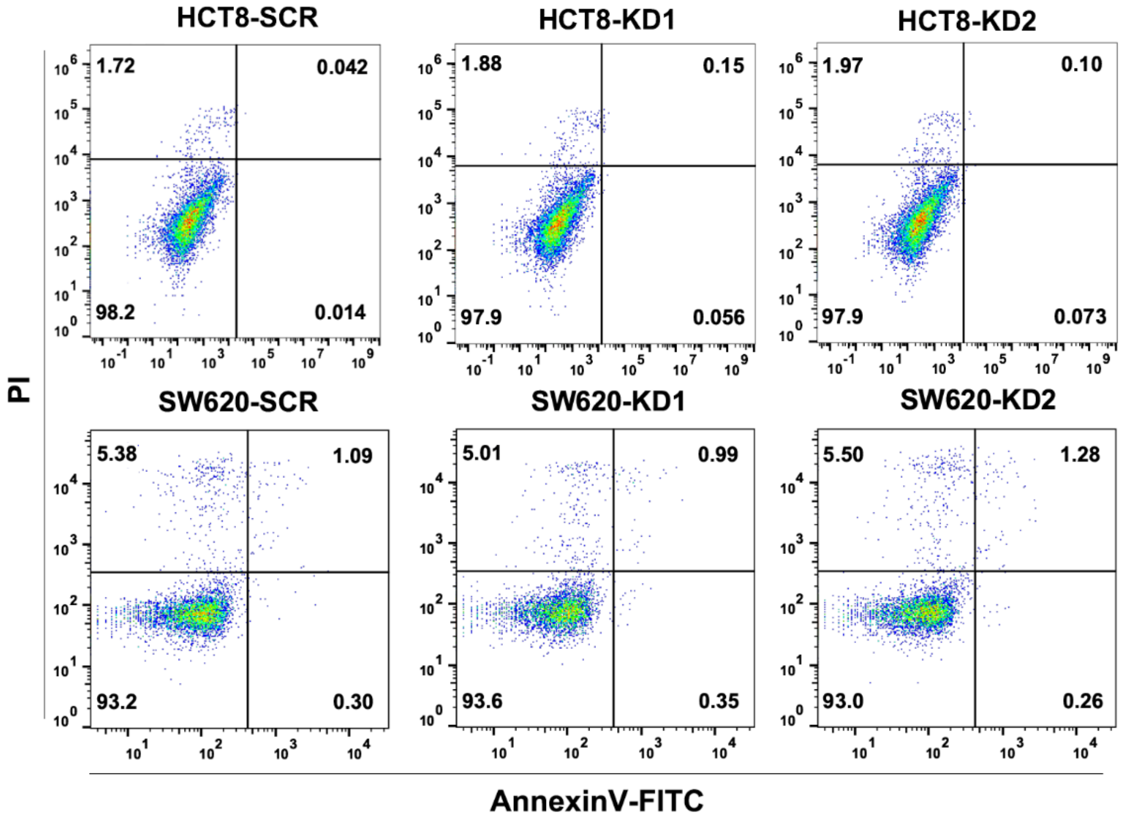

Figure. S2 Knocking down DDX21 does not affect apoptosis. Flow cytometry analyses the effects of DDX21 blockade on HCT8 and SW620 cell apoptosis.

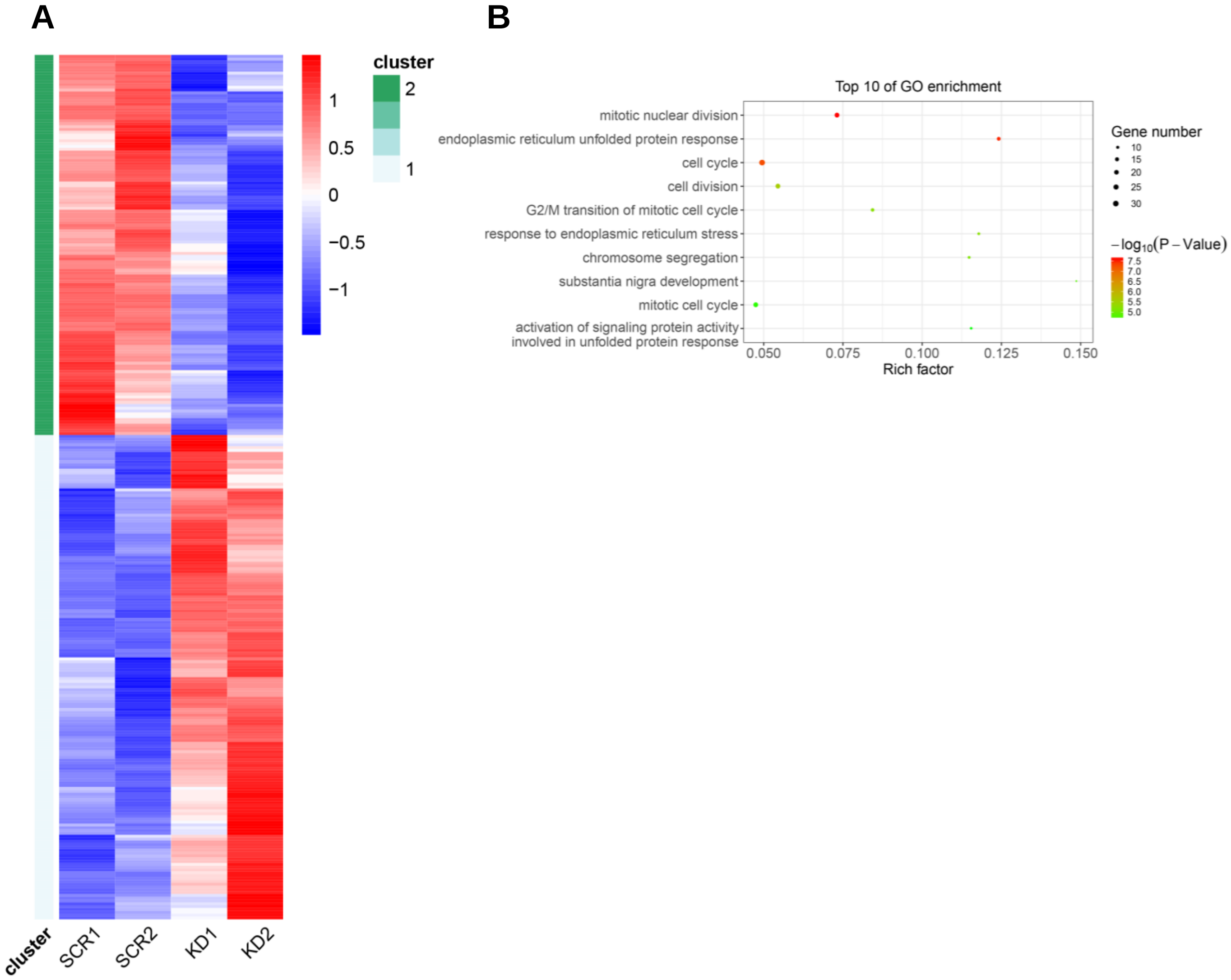

Figure. S3 RNAseq analysis of DDX21 knocking down cells. A, Heatmap of altered genes in DDX21 down expressed HCT8 cells compared with control cells. B, GO analysis for all genes with altered expressions, shows top 10 enrichment.

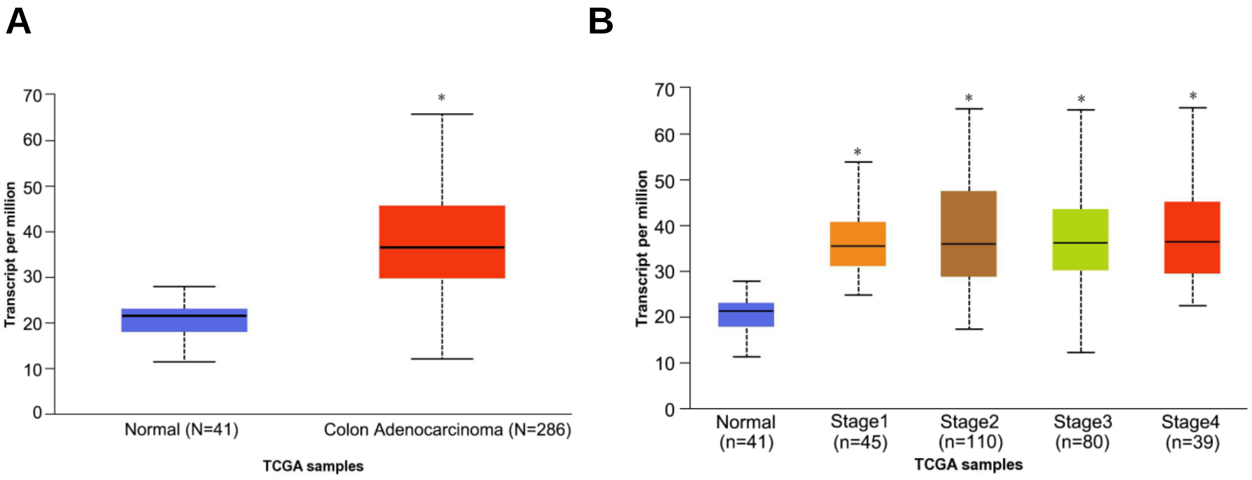

Figure. S4 WDR5 is overexpressed in CRC. WDR5 is highly expressed in colon adenocarcinoma compared with normal tissues. A, and is overexpressed from stage 1 to stage 4. B, Analysis was performed by UALCAN. *$P < 0.05$.

Table S1 Primers for mRNA qRT-PCR

Gene Symbol	Forward (5'-3')	Reverse (5'-3')
GAPDH	GAAGGTGAAGGTCGGAG	GAAGATGGTGATGGGATTTC
DDX21	TCATCAAGGACGCACTATCATCT	CCTTTCAGGGTGATTTCCCTTT
CDK1	AAACTACAGGTCAAGTGGTAGCC	TCCTGCATAAGCACATCCTGA
GMNN	AAAACGGAGAAAGGCGCTGTA	GGCGGGCAATTTCATTGTCC
NUSAP1	AGCCCATCAATAAGGGAGGG	ACCTGACACCCGTTTTAGCTG
CCNA2	TGGAAAGCAAACAGTAAACAGCC	GGGCATCTTCACGCTCTATTT
CEP55	AGTAAGTGGGGATCGAAGCCT	CTCAAGGACTCGAATTTTCTCCA
NEK2	TGCTTCGTGAACTGAAACATCC	CCAGAGTCAACTGAGTCATCACT
PTTG1	ACCCGTGTGGTTGCTAAGG	ACGTGGTGTTGAAACTTGAGAT
DLGAP5	AAGTGGGTCGTTATAGACCTGA	TGCTCGAACATCACTCTCGTTAT
CCNB1	AATAAGGCGAAGATCAACATGGC	TTTGTTACCAATGTCCCCAAGAG
AURKA	GAGGTCCAAAACGTGTTCTCG	ACAGGATGAGGTACACTGGTTG
TPX2	ATGGAACTGGAGGGCTTTTTC	TGTTGTCAACTGGTTTCAAAGGT
CDCA2	TGCCGAATTACCTCCTAATCCT	TGCTCTACGGTTACTGTGGAAA
NES	GAAGGGCAATCACAACAGGTG	GGGGCCACATCATCTTCCA
BUB1	TGGGAAAGATACATACAGTGGGT	AGGGGATGACAGGGTTCCAAT
FEN1	ATGACATCAAGAGCTACTTTGGC	GGCGAACAGCAATCAGGAACT
HJURP	GATTCAAAAAGCGGTGAGGTCG	CTCACCGCTTTTTGAATCGGC
NCAPD3	TGGTGTCCGCTGGATCTTAGA	AGTCACACGTACATCCCTTCC
SPAG5	TTGAGGCCCGTTTAGATACCA	GCTTTCCTTGGAGCAATGTAGTT
CDT1	CGGTGGACGAGGTTTCCAG	CGGTGGACGAGGTTTCCAG
CCND1	GCTGCGAAGTGGAAACCATC	CCTCCTTCTGCACACATTTGAA

Table S2 Antibodies

Name	Source	Identifier
Rabit monoclonal anti-DDX21	abcam	ab182156
Rabbit polyclonal anti-WDR5	proteintech	15544-1-AP
Rabbit polyclonal anti-CDK1	proteintech	19532-1-AP
Rabbit polyclonal anti-H3K4me3	abcam	ab8580
Rabbit polyclonal anti-HSP70	ABclonal	A0284
Mouse monoclonal anti-GAPDH	proteintech	HRP-60004
Rabit monoclonal anti-ACTB	ABclonal	AC026
Rabbit polyclonal anti-Flag	proteintech	20543-1-AP

Table S3 Primers for ChIP qRT-PCR

**Primer of CDK1 Promoter**	**Forward (5'-3')**	**Reverse (5'-3')**
P1	TCCCAATTATCATCCTGCTCC	GCTATCATCTTGGCAAATACAGT
P2	CACCAGATGATAGATGCAGCTC	CCCAAATCAGGATATGGAATGGA
P3	ACTGCAGTAAGTGCAGAAATCTC	ACCACGCCTGGCTTAAATATAATT
P4	TGTTCGCTCCGTTCTTCT	ACTATACACTCCTAACCCTAA
P5	GCGTAGCTGGGCTCTGATT	GCCACTGTACCCGGCTTATT
P6	GCGGAGTAGCAGGCTCTTTC	CACGAGTTCCAGGATCGGTTT
